# Dental Personnel Treated for Idiopathic Pulmonary Fibrosis at a Tertiary Care Center — Virginia, 2000–2015

**DOI:** 10.15585/mmwr.mm6709a2

**Published:** 2018-03-09

**Authors:** Randall J. Nett, Kristin J. Cummings, Brenna Cannon, Jean Cox-Ganser, Steven D. Nathan

**Affiliations:** ^1^Respiratory Health Division, National Institute for Occupational Safety and Health, CDC; ^2^Inova Fairfax Hospital, Falls Church, Virginia.

In April 2016, a Virginia dentist who had recently received a diagnosis of idiopathic pulmonary fibrosis (IPF) and was undergoing treatment at a specialty clinic at a Virginia tertiary care center contacted CDC to report concerns that IPF had been diagnosed in multiple Virginia dentists who had sought treatment at the same specialty clinic. IPF is a chronic, progressive lung disease of unknown cause and associated with a poor prognosis (*1*). Although IPF has been associated with certain occupations (*2*), no published data exist regarding IPF in dentists. The medical records for all 894 patients treated for IPF at the Virginia tertiary care center during September 1996–June 2017 were reviewed for evidence that the patient had worked as a dentist, dental hygienist, or dental technician; among these patients, eight (0.9%) were identified as dentists and one (0.1%) as a dental technician, and each had sought treatment during 2000–2015. Seven of these nine patients had died. A questionnaire was administered to one of the living patients, who reported polishing dental appliances and preparing amalgams and impressions without respiratory protection. Substances used during these tasks contained silica, polyvinyl siloxane, alginate, and other compounds with known or potential respiratory toxicity. Although no clear etiologies for this cluster exist, occupational exposures possibly contributed. This cluster of IPF cases reinforces the need to understand further the unique occupational exposures of dental personnel and the association between these exposures and the risk for developing IPF so that appropriate strategies can be developed for the prevention of potentially harmful exposures.

IPF is a form of chronic, progressive fibrosing interstitial pneumonia of unknown cause. IPF is associated with histopathologic and radiologic patterns of usual interstitial pneumonia in the absence of other known causes of interstitial lung disease (*1*) and is characterized by unexplained slowly progressive dyspnea that can be accompanied by a nonproductive cough (*2*). Available treatment options for IPF include pharmacotherapy (i.e., pirfenidone and nintedanib) and lung transplantation (*2*). The estimated median survival after diagnosis is 3–5 years (*2*). Although the etiology of IPF is unknown, exposures that have been suggested as contributing factors include viral infections, cigarette smoking, and occupations where exposure to dust, wood dust, and metal dust are common (*2*). In the United States, on the basis of the case definitions used by separate studies to analyze data collected during 1988–2005, the estimated annual incidence of IPF varied from 6.8 to 17.4 per 100,000 population, and the estimated prevalence varied from 14.0 to 63.0 per 100,000 population (*3*) and increased with increasing age (*2*). No published data could be found regarding dental personnel and IPF.

In June 2017, the electronic medical records of all 894 patients with a diagnosis of IPF treated at the Virginia specialty clinic during September 1996–June 2017 were reviewed to identify patients having the occupation of dentist, dental hygienist, or dental technician. Available electronic medical records of patients identified as having one of these occupations were reviewed, pertinent data were abstracted, and an attempt was made to interview living patients to ascertain symptoms and occupational and nonoccupational exposures, after obtaining informed consent. This study received approval from the Inova Fairfax Hospital Institutional Review Board.

Among 894 patients treated for IPF at the tertiary care center, nine (1%) were identified as dental personnel, including eight dentists and one dental technician. All patients were male and were treated during 2000–2015. Five were white, one was black, and the race of three was unknown. At the time of pulmonary consultation, the median patient age was 64 years (range = 49–81 years) (Table). States of residence included Virginia (five), Maryland (three), and Georgia (one). Seven of the nine patients had died; among these, the median survival time from consultation was 3 years (range = 1–7 years). Among eight patients tested at the time of pulmonary consultation, pulmonary function tests demonstrated three patients had normal spirometry, two of whom also had documented normal lung volumes, and five patients had restrictive spirometry and low lung volumes, interpreted as lung restriction. Each of the five patients with restriction had low predicted values for diffusing capacity of the lungs for carbon monoxide (D_LCO_) (median = 47% [range = 19%–55%]). Pulmonary function test results were not available for one patient. One of the living patients who did not complete an interview underwent a lung transplant 3 years after diagnosis. No tissue specimens were available for analysis.

**TABLE:** Selected characteristics of nine male dental personnel treated for idiopathic pulmonary fibrosis at time of first pulmonary consultation at a tertiary care center — Virginia, 2000–2015

Age (yrs)	Symptoms	Tobacco use	Pulmonary function*^,†^	Computed tomography finding	Clinical follow-up
49	NA	NA	Moderate restriction	Extensive basilar honeycombing	Died 1 year after initial consultation
50	Cough, phlegm, SOB	NA	Severe restriction	Extensive honeycombing and traction bronchiectasis	Alive. Underwent lung transplant 3 years after diagnosis.
58	DOE	Former	Mild restriction	Basilar subpleural fibrosis, diffuse peripheral septal thickening with cystic changes	Died 7 years after initial consultation
63	SOB	Former	Normal^§^	Advanced fibrosis, honeycombing, bullous and cystic lesions	Died 3 years after initial consultation
64	NA	NA	NA	Peripheral reticular infiltrates	Died 3 years after initial consultation
66	NA	NA	Restriction^¶^	Bibasilar infiltrates, bibasilar honeycombing	Died 6 years after initial consultation
70	DOE, decreased exercise tolerance	Former	Normal	NA	Died 4 years after initial consultation
70	Cough, throat clearing, SOB	Never	Mild restriction	NA	Alive
81	NA	NA	Normal	Mild to moderate subpleural fibrosis with bibasilar honeycombing	Died 2 years after initial consultation

**Abbreviations:** DOE = dyspnea on exertion; NA = not available; SOB = shortness of breath.

* Interpretation conducted by investigators and based on spirometry and measurement of total lung capacity, when available.

^†^ Severity classified using criteria established by the American Thoracic Society/European Respiratory Society Task Force (http://www.thoracic.org/statements/resources/pft/pft5.pdf).

^§^ Only spirometry results available.

^¶^ Severity cannot be classified because forced expiratory volume in 1 second not available.

Three patients were former smokers, one had never smoked, and smoking history was unknown for five (Table). A telephone interview was conducted with the patient who had contacted CDC; it was not possible to complete an interview with the other living patient. The interviewed patient, who had never smoked, reported not wearing a National Institute for Occupational Safety and Health-certified respirator during dental activities throughout his 40-year dental practice; he wore a surgical mask for the last 20 years of his dental practice. He reported performing polishing of dental appliances, preparing amalgams and impressions, and developing x-rays using film developing solutions. He also reported work-related exposure to dust while working as a street sweeper for 3 months before entering dental school and environmental exposure to dust from coral beaches for approximately 15 years while intermittently visiting the Caribbean region as a practicing dentist.

## Discussion

During September 1996–June 2017, nine (1%) of 894 patients treated for IPF at a single tertiary care center in Virginia were identified as dental personnel. Each patient presented for care during 2000–2015. Seven of the patients had died. This is the first known described cluster of IPF occurring among dental personnel. Although no clear etiology exists for this cluster, it is possible that occupational exposures contributed to the development of IPF.

During 2016, dentists accounted for an estimated 0.038% of U.S. residents (*4*), yet represented 0.893% of patients undergoing treatment for IPF at one tertiary care center, nearly a 23-fold difference. Dental personnel are exposed to infectious agents, chemicals, airborne particulates, ionizing radiation, and other potentially hazardous materials (*5*). Inhalational exposures experienced by dentists likely increase their risk for certain work-related respiratory diseases. For example, cases of dental technicians with pneumoconiosis, a restrictive occupational lung disease resulting from inhalation of dust, have been identified after exposure to either silica or cobalt-chromium-molybdenum-based dental prostheses (*6*,*7*). A case of pneumoconiosis was identified postmortem in an elderly dentist who died from respiratory failure (*8*). Examination of lung tissue at autopsy using scanning electron microscopy revealed particles consistent with alginate impression powders used during the dentist’s practice. Nine cases of silicosis were recognized among dental laboratory technicians exposed to crystalline silica in five states during 1994–2000 (*9*). Asbestos-related lung disease, attributed to manipulating wet asbestos-containing paper during preparation of molds in casting operations, has also been identified in dentists (*10*). The one living patient in this cluster who was interviewed reported occupational exposures to silica and other materials used in dental practice, but also other work-related and environmental exposures to dust.

IPF has not been previously described among dental personnel. However, a query of the National Occupational Respiratory Mortality System for 4 separate years (1999, 2003, 2004, and 2007) for “other interstitial pulmonary diseases with fibrosis”* (which would include IPF) listed as the underlying or contributing cause of death revealed 35 decedents categorized as having worked in the “office of dentists” and 19 categorized as having the occupation “dentist,” with proportionate mortality ratios of 1.52 (95% confidence interval [CI] = 1.05–2.11) and 1.67 (95% CI = 1.01–2.61), respectively (Respiratory Health Division, CDC, unpublished data, 2017). These findings suggest that a higher rate of IPF might occur among dental personnel than among the general population.

The living patient who was interviewed reported occupational exposures to known respiratory hazards (e.g., silica) yet did not wear National Institute for Occupational Safety and Health-certified respiratory protection. It is possible other patients in this case series had similar experiences. Dental personnel who perform tasks that result in occupational exposures to known respiratory hazards should wear adequate respiratory protection if other controls (e.g., improved ventilation) are not practical or effective (https://www.osha.gov/SLTC/respiratoryprotection/index.html). If respiratory protection is used, a written respiratory protection program should be implemented as required by the Occupational Safety and Health Administration Respiratory Protection Standard, including training, fit testing, and maintenance and use requirements (https://www.osha.gov/pls/oshaweb/owadisp.show_document?p_id=12716&p_table=STANDARDS).

The findings in this report are subject to at least four limitations. First, in this analysis, only patients undergoing treatment at a single tertiary care center specializing in IPF treatment were identified, which might have led to an overrepresentation of dentists, given their comparatively high socioeconomic status. Conversely, dental personnel in Virginia and the surrounding region undergoing IPF treatment at other facilities during this same time frame were not identified, thereby potentially underrepresenting the magnitude of this cluster. Second, only one patient completed an interview, limiting the ability to explore past occupational exposures. Third, multiple patients had reported exposures that occurred outside of work and that are known risk factors for IPF, including tobacco smoke and dust (*2*). Finally, no biopsy specimens were available for examination to assess histological commonalities among the patients.

This investigation revealed the first described cluster of dental personnel with diagnosed IPF. The eight dentists identified in this cluster exceeded the number of expected cases, consistent with National Occupational Respiratory Mortality System data regarding IPF mortality and the proportion of U.S. residents who are dentists. Dentists and other dental personnel experience unique occupational exposures, including exposure to infectious organisms, dusts, gases, and fumes. It is possible that occupational exposures contributed to this cluster. After this analysis, another IPF case was diagnosed in a dentist treated at this specialty clinic. Further investigation of the risk for dental personnel and IPF is warranted to develop strategies for prevention of potentially harmful exposures.

**Summary**

**What is already known about this topic?**

Idiopathic pulmonary fibrosis (IPF) is a chronic, progressive lung disease of unknown cause and is associated with a poor prognosis. IPF has been associated with certain occupations; however, no published data exist regarding IPF in dental personnel.

**What is added by this report?**

A unique cluster of nine cases of IPF was identified among dental personnel treated at a tertiary care center in Virginia during 2000–2015. No clear etiology has been identified, but occupational exposures are possible.

**What are the implications for public health practice?**

During 2016, approximately 650,000 dental personnel were estimated to be employed in the United States, including 122,330 dentists. This cluster of IPF cases reinforces the need to understand further the occupational exposures of dental personnel and the association between these exposures and the risk for developing IPF so that strategies can be developed for prevention of potentially harmful exposures.

## Footnotes

* *International Classification of Diseases, Tenth Revision* (ICD-10) code J84.1.
